# Advancing surgical frontiers: endorobotic submucosal dissection for enhanced patient outcomes

**DOI:** 10.1007/s10151-024-03009-y

**Published:** 2024-10-04

**Authors:** A. Ulkucu, A. Kaya, T. Schwenk, S. Elsoukkary, E. Gorgun

**Affiliations:** 1 Department of Colorectal Surgery, Digestive Disease and Surgery Institute, Cleveland Clinic, Cleveland, OH USA; 2 Department of Pathology, Pathology & Laboratory Medicine Institute, Cleveland Clinic, Cleveland, OH USA

Endoscopic submucosal dissection (ESD) represents a crucial advancement in minimally invasive gastroenterological interventions, enabling precise excision of early neoplasms [1, 2]. However, its technical intricacy and steep learning curve impede widespread implementation [3, 4]. Endorobotic submucosal dissection (ERSD) emerges as a solution, incorporating robotic technology to enhance dexterity and providing superior three-dimensional visualization, potentially mitigating ESD's limitations [5].

A 75-year-old male with a significant sigmoid colon lesion underwent ERSD. The GelPOINT Path® (Applied Medical Resources Corp., Rancho Santa Margarita, CA), a flexible ring-shaped transanal access platform positioned at the anal verge, enables pneumorectum establishment and enhanced instrument access. The Da Vinci SP (Intuitive Surgical, Sunnyvale, CA) surgical system was employed, which deploys articulating instruments and a 3D camera through a single 25-mm port. Meticulous submucosal dissection was performed utilizing the robotic system's precision, with a spatula facilitating controlled dissection in the submucosal plane. Complete lesion excision was achieved.

The postoperative course was uneventful, with the patient transferred to recovery in stable condition. Histopathology confirmed a tubulovillous adenoma exhibiting high-grade dysplasia, with dysplasia-free cauterized margins. This case exemplifies ERSD's efficacy in managing complex colonic lesions, demonstrating its potential for optimizing outcomes through minimally invasive techniques and its adaptability in colonic navigation.

This case demonstrates ERSD's advancement in minimally invasive surgery, integrating robotic technology to enhance dexterity and 3D visualization. Utilizing the Da Vinci SP system and GelPOINT Path platform showcases technical progress, enabling precise dissection and improved navigation. The emphasis on clear margins in histopathology underscores its prognostic importance. Overall, this exemplifies surgical evolution towards improved outcomes via technological innovation.

## Supplementary Information

Below is the link to the electronic supplementary material.

Supplementary file1 (MP4 299018 KB)

## Data Availability

No datasets were generated or analysed during the current study.
